# Buffalo Medical and Surgical Association

**Published:** 1883-04

**Authors:** Clayton M. Daniels

**Affiliations:** Secretary


### Society Reports.

BUFFALO MEDICAL AND SURGICAL ASSOCIATION.

*Regular Meeting, March 6th, 1883.*

Vice-President Dr. John Cronyn in the Chair. Dr. C. M. Daniels, Secretary.

Present—Drs. Fell, Dayton, Bartlett, Peterson, Harvey, Clark, Mann, Howe, C. A. Ring, Fowler, Burghardt, Runner, Potter,

Brecht, and by invitation, Drs. Hayd, F. H. Potter and Tryon.

*Essay*—By Dr. Julius Pohlman; Subject, "Geology in Sanitary Science." After referring to the various specialties in medicine and of the specialists in naturally finding the cause of disease in each of their several departments, he especially referred to "geology," his favorite study, and of its importance in relation to the laws of health, and gave a very clear and comprehensive explanation of Buffalo and its situation in a sanitary point of view, and in detail especially dwelt upon the unhealthfulness of the eastern portions of the city, where the soil is an almost impervious clay and where sewers are wanting, and the people built their houses over stagnant water and live in utter disregard to drainage or surroundings. He thought it too late to try and educate the people to observe the necessities for drainage, as they were becoming accustomed to the present condition of things and would make no effort for their own relief. Compulsion only would avail, together with a proper system of general sewerage.

*Discussion*—Dr. Bartlett thought that the essayist did not understate the facts, especially regarding the conditions under many of the houses, and thought the builders themselves should give more attention to the matter. Related a case where he was asked to see a family where five persons died from typhoid fever. Examination of the premises discovered a considerable quantity of stagnant water under the house, and the warm rooms above naturally drew the poisonous emanations up through them. He wished the law could be made to force the necessary sanitary requirements.

Dr. Hayd thought that the facts placed before the association were such that the physicians should urge the authorities to take them up, and that it fully illustrated that many diseases were caused or promoted by the bad sanitary arrangements; also, that the paper should be published for the benefit of the public.

Dr. Howe testified to his appreciation of the subject, but thought we should be guarded in regard to certainty in ascribing the effect to an apparent cause, for sometimes the most perfect types of health came from some of the poorest dwellings. As to the health department, he thought its powers had recently been improved.

Dr. C. A. Ring asked regarding the drainage in the vicinity of the almshouse, its natural direction, etc.

Dr. Pohlman replied that it was south towards the city, but that he could not advise an effort to force it in that direction, although, no doubt, a large portion flows down into the earth through many of the deep fissures in the rocks before reaching the city itself.

Dr. Fell remarked that papers of this character should come more frequently before the association, and not allowed to die out as soon as read, and thought the Board of Health should be given greater powers; also spoke of the fallacy of supposing that water which is clear and cold is necessarily pure, and related a case where water which seemed of the best came from direct sewer contamination. In speaking of the low clay soil in the fifth ward, as referred to by the essayist, he said in summer it was doing injustice to the "Hamburg canal," to invite the comparison. As to the action of the authorities in doing away with the wells, he thought it a wise and proper act. As to the necessity for sewers at East Buffalo, he thought the city should build them at once.

Dr. Fowler spoke of the mortality in the fifth ward, as taken by himself some years ago, and although the ward contained nearly one-fourth of the total population of Buffalo, the death-rate at that time did not exceed that of any other portion of the city.

Dr. Clark thought the subject one of great importance and that it was of great interest to the public, and seconded Dr. Hayd in recommending that the paper be published.

Dr. Bartlett, referring to Dr. Fowler's statistics, thought that at the time there might have been what would be a standing center of disease outside of the fifth ward, thereby affecting the comparison.

Dr. Fowler replied that the statistics taken covered a period of twenty-seven months.

Dr. Cronyn said that the association and community could not help but thank the essayist for his able and timely paper upon a subject of the greatest importance to all. It was easy to tell the people they were sick because of the cesspool near by in the shape of wells, etc. He had preached it in Buffalo for twenty-three years and insisted that well water should not be used. He considered the paper the best and most important one he had heard upon the subject.

Dr. Fowler moved a vote of thanks be tendered Dr. Pohlman. Carried.

*Voluntary Communications*—Dr. Howe presented a picture of typical form of strumous diathesis.

Dr. Mann presented a sack of an ovarian cyst, removed during the day, the tumor weighing sixty pounds, and stated that the ordinary weight of the woman was but ninety pounds.

*Prevailing Diseases*—Dr. Bartlett reported pneumonia and a peculiar form of modified influenza, which seemed, to a certain extent, to be transmissible; very little diphtheria, also scarlet fever, not especially severe. Dr. Fowler had observed that which Dr. Bartlett reported; also some evidences of broncho-pneumonia. Dr. Brecht some diphtheria.

*Miscellaneous Business*—Dr. Mann announced that the new Medical Library Association was quartered with Dr. Samo, corner of Pearl and Chippewa streets, and stated that the library of the Erie County Medical Society was also there, and that a large number of journals and periodicals were now on file and the library open from two until ten o'clock P. M. daily.

Adjourned. CLAYTON M. DANIELS, *Secretary.*

